# Impact of sarcopenic obesity on post-hepatectomy bile leakage for hepatocellular carcinoma

**DOI:** 10.1371/journal.pone.0286353

**Published:** 2023-10-05

**Authors:** Hikaru Hayashi, Akira Shimizu, Koji Kubota, Tsuyoshi Notake, Hitoshi Masuo, Takahiro Yoshizawa, Kiyotaka Hosoda, Hiroki Sakai, Koya Yasukawa, Yuji Soejima

**Affiliations:** Division of Gastroenterological, Hepato-Biliary-Pancreatic, Transplantation and Pediatric Surgery, Department of Surgery, Shinshu University School of Medicine, Nagano, Japan; Humanitas Clinical and Research Center - IRRCS, ITALY

## Abstract

**Background:**

Post-hepatectomy bile leakage (PHBL) is a potentially fatal complication that can arise after hepatectomy. Previous studies have identified obesity as a risk factor for PHBL. In this study, we investigated the impact of sarcopenic obesity on PHBL in hepatocellular carcinoma (HCC) patients.

**Methods:**

In total, we enrolled 409 patients who underwent hepatectomy without bilioenteric anastomosis for HCC between January 2010 and August 2021. Patients were grouped according to the presence or absence of PHBL. Patient characteristics, including body mass index and sarcopenic obesity, were then analyzed for predictive factors for PHBL.

**Results:**

Among the 409 HCC patients included in the study, 39 developed PHBL. Male sex, hypertension, cardiac disease, white blood cell counts, the psoas muscle area, and visceral fat area, and intraoperative blood loss were significantly increased in the PHBL (+) group compared with the PHBL (−) group. Multivariate analysis showed that the independent risk factors for the occurrence of PHBL were intraoperative blood loss ≥370 mL and sarcopenic obesity.

**Conclusions:**

Our results show that it is important to understand whether a patient is at high risk for PHBL prior to surgery and to focus on reducing intraoperative blood loss during surgery for patients with risk factors for PHBL.

## Introduction

Post-hepatectomy bile leakage (PHBL) is one of the most common and notable complications after hepatectomy, occurring in approximately 5−10% of patients [1, 2], and can lead to surgical site infection (SSI) or post-hepatectomy liver failure (PHLF) [3]. According to previous reports, factors such as liver cirrhosis, non-anatomical hepatectomy, and obesity have been identified as risk factors for PHBL [4, 5].

Recently, sarcopenic obesity has been reported to be associated with postoperative outcomes in various carcinomas. Kim et al. [6] showed that sarcopenic obesity was an independent risk factor for increased mortality in patients with gastric cancer. Kobayashi et al. [7] reported that sarcopenic obesity was a significant prognostic factor for poor overall survival and relapse-free survival after hepatectomy for hepatocellular carcinoma (HCC). However, few studies have reported the relationship between short-term outcomes, especially PHBL, and sarcopenic obesity after hepatectomy for HCC.

The purpose of this study was to evaluate the impact of sarcopenic obesity on the occurrence of PHBL after hepatectomy for HCC.

## Materials and methods

In total, 409 patients who underwent hepatectomy without bilioenteric anastomosis for HCC between January 2010 and August 2021 were enrolled in this study. Patients were categorized into the following two groups based on the presence or absence of PHBL: PHBL (+) and PHBL (−). Patient characteristics and relevant clinicopathological variables, surgical details, and short-term outcomes were recorded. The study was approved by the Biological and Medical Research Ethics Committee of Shinshu University School of Medicine (approval no. 5701) and was conducted in accordance with the principles of the Declaration of Helsinki. Owing to the retrospective nature of the study and the absence of invasive interventions, the requirement for written consent was waived by the review board, and consent was obtained through an opt-out method. The data were collected between December 2022 and March 2023 and analyzed anonymously by several physicians on the basis of medical records.

### Perioperative management

In this study, the hepatectomy procedures were conducted by several surgeons. Parenchymal transection was performed using an ultrasonic dissector and/or a clamp-crushing technique. The intermittent Pringle maneuver (PM) was routinely used to control intraoperative blood loss for 15 min, followed by 5 min of reperfusion; this process was repeated as needed. Abdominal drains were routinely placed along the cut surface of the liver. Postoperatively, bilirubin concentrations in the drainage fluid were routinely measured twice per week for surveillance of bile leakage. In addition, for several days after surgery, the presence or absence of fluid collection around the hepatectomy site was checked every day by ultrasonography. When PHBL was detected, additional percutaneous drainage or exchange of drainage tubes was performed as needed. The end of follow‐up was December 2021 or the time of discharge.

### Definitions

Pathological findings were evaluated in accordance with the American Joint Committee on Cancer (AJCC) Staging Manual, 7th edition [8]. Liver cirrhosis was defined as a fibrosis score of 4 using the new Inuyama classification [9]. Postoperative complications were graded using the Clavien–Dindo classification [10]. PHLF and PHBL were diagnosed and graded according to the criteria of the International Study Group of Liver Surgery [11, 12]. Major hepatectomy was defined as resection of three or more Couinaud segments of the liver. Anatomical resection included Couinaud segmentectomy, sectionectomy, hemihepatectomy, and trisectionectomy.

**Fig 1:**
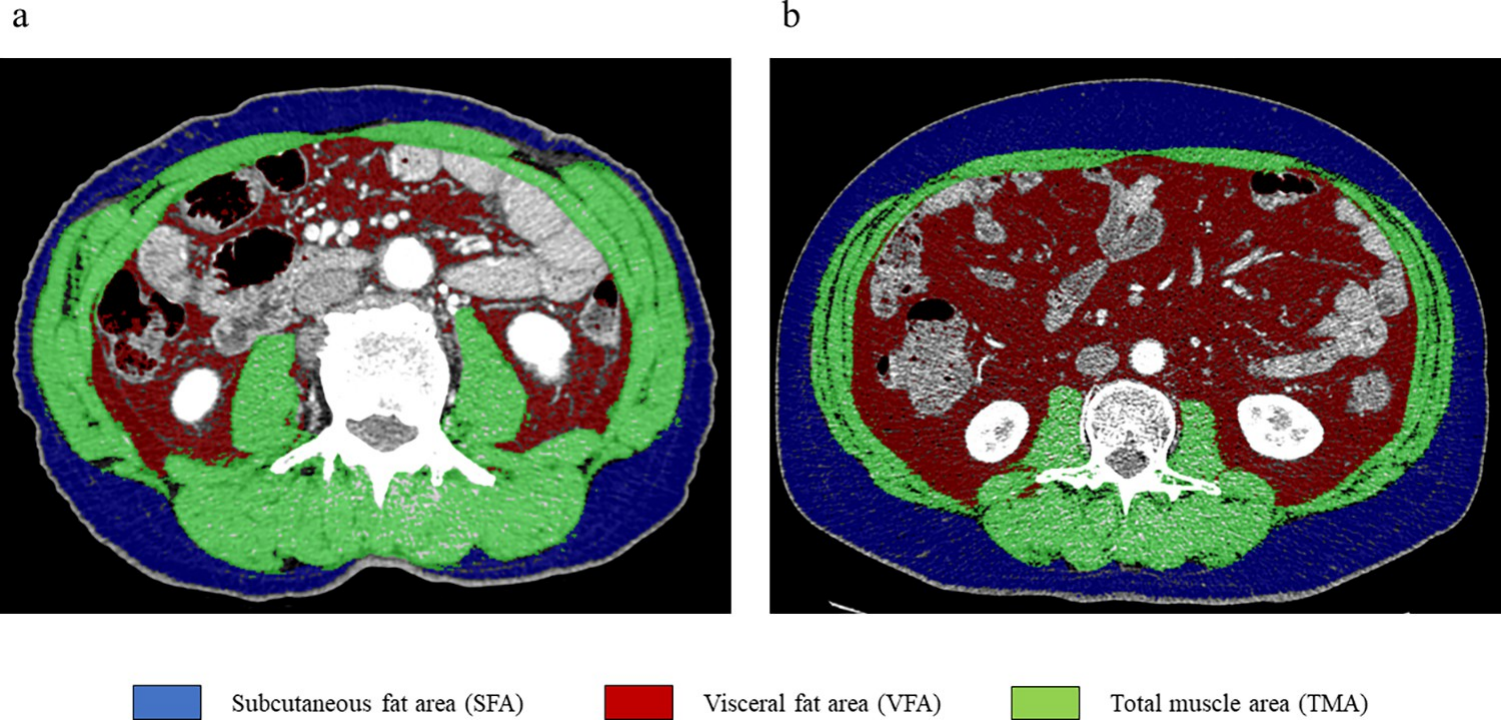
Computed tomography image analysis of a third lumbar vertebra. Green, muscle area; red, visceral fat area; blue, subcutaneous fat area. (a) A patient without sarcopenic obesity. (b) A patient with sarcopenic obesity.

Multidetector-row computed tomography (CT) was performed within 4 weeks before surgery for diagnostic and staging purposes and was used to evaluate sarcopenia, which was defined in accordance with the international consensus [13] as a skeletal muscle index (SMI) of <52.4 cm^2^/m^2^ for men and <38.9 cm^2^/m^2^ for women. SMI was defined as the total muscle area measured on an axial section through the third lumbar vertebra (L3) where both pedicles were visible with a preestablished density threshold of −29 to +150 Hounsfield units normalized for stature (Fig 1). The visceral fat area (VFA) and subcutaneous fat area were also measured, and sarcopenic obesity was defined as the VFA/SMI ratio [14, 15]. The cut-off value for sarcopenic obesity was defined in accordance with the maximum sensitivity and specificity for predicting PHBL in a receiver operating characteristic (ROC) curve analysis; the cut-off value was 1.65. The CT scans were checked by two qualified physicians using a Synapse Vincent FN-7941 (Fujifilm, Tokyo, Japan).

### Statistical analysis

All data were collected by a research assistant and stored in a computer database. Statistical analyses were performed by the chi-square test or Fisher’s exact test to compare categorical variables and by the Mann–Whitney U test to compare continuous variables. ROC curve analyses were carried out for the predictive parameters to evaluate the associations with PHBL, and the Youden index was used to determine the cut-off values. Multivariate analysis using a logistic regression model was conducted to identify significant predictive factors for the occurrence of PHBL. All statistical analyses were performed using JMP Pro 16.2 (SAS Institute Inc., Cary, NC, USA).

## Results

### Patient characteristics

The study involved 409 patients who underwent hepatectomy. Among the total patients, 39 (9.5%) developed PHBL. The background characteristics and comorbidities of the patients according to the presence or absence of PHBL are summarized in Table 1. The ratio of male sex and the prevalences of hypertension and cardiac disease were higher in the PHBL (+) group than in the PHBL (−) group (89.7% vs. 74.3%; 74.4% vs. 53.0%; 28.2% vs. 13.0%; *P* = 0.020, 0.009, and 0.018, respectively). The indocyanine green retention rate at 15 min was lower in the PHBL (+) group than in the PHBL (−) group (9.7% vs. 12.0%; *P* = 0.026). No significant differences were observed between the two groups for other variables, including age, etiology, and Child–Pugh classification.

**Table 1:** Patient characteristics and surgical outcomes.

Variables	PHBL (+) (*n* = 39)		PHBL (−) (*n* = 370)		*P* Value
*Characteristics*					
Age, years ^a^	71	(44–82)	71	(36–89)	0.977
Sex, male/female	35	/4	275	/95	0.020
Body mass index, kg/m^2^ ^a^	23.1	(20.2–31.6)	22.9	(13.4–45.2)	0.168
Etiology					
HBsAg positive	5	(13)	80	(22)	0.175
HCVAb positive	12	(31)	151	(41)	0.216
Social history					
Smoking	29	(74)	236	(64)	0.179
Drinking	32	(82)	281	(76)	0.379
Comorbidity					
Hypertension	29	(74)	196	(53)	0.009
Diabetes mellitus	14	(36)	121	(33)	0.688
Cardiac disease	11	(28)	48	(13)	0.018
Pulmonary disease	2	(5)	40	(11)	0.227
Child-Pugh classification					0.203
Grade A	39	(100)	362	(98)	
Grade B	0	(0)	8	(2)	
Laboratory data					
Albumin, g/dl^a^	4.1	(3.3–5.1)	3.9	(2.9–5.2)	0.161
T-Bil, mg/dl^a^	0.79	(0.3–2.22)	0.80	(0.2–2.81)	0.384
AST, units/l^a^	24	(11–74)	30	(11–142)	0.005
ALT, units/l^a^	29	(6–149)	30	(7–215)	0.278
PT%^a^	86.0	(53.4–109.7)	87.1	(28.1–146.6)	0.322
White blood cell, /μl^a^	5090	(2450–11220)	4275	(1770–10610)	0.032
Platelet, ×10^4^ /μl^a^	15.3	(4.7–25.2)	14.3	(4.0–45.6)	0.158
Tumor markers					
AFP > 20 ng/ml	15	(38)	135	(36)	0.808
PIVKA-II > 40 mAU/ml	20	(51)	178	(48)	0.706
Modified ALBI grade, 1 / 2a / 2b / 3	25	/ 10 / 4 / 0	184	/ 124 / 62 / 0	0.213
ICGR15, %^a^	9.7	(2.9–26.3)	12.0	(2.6–46.4)	0.026
Sarcopenia	16	(41)	141	(38)	0.723
Sarcopenic obesity	35	(90)	221	(60)	< 0.001
Parameters associated with muscle and fat area at the level of third lumbar vertebra					
Skeletal muscle index, cm^2^/m^2^ ^a^	53.3	(38.1–71.4)	51.2	(32.2–85.4)	0.477
Total muscle area, cm^2^ ^a^	143.8	(83.5–221.2)	135.8	(77.3–234.0)	0.150
Psoas muscle area, cm^2^ ^a^	16.6	(4.1–27.4)	14.0	(4.9–31.4)	0.016
Visceral fat area, cm^2^ ^a^	135.3	(40.8–254.5)	96.8	(2.6–330.9)	0.003
Subcutaneous fat area, cm^2^ ^a^	107.4	(46.9–251.9)	100.2	(3.6–347.4)	0.141
*Surgical outcomes*					
Type of hepatectomy					0.373
Partial hepatectomy	16	(41)	187	(51)	
Segmentectomy	8	(21)	84	(23)	
Sectionectomy	11	(28)	61	(16)	
Hemihepatectomy or more	4	(10)	38	(10)	
Primary hepatectomy	30	(77)	272	(74)	0.641
Major hepatectomy	4	(10)	48	(13)	0.619
Anatomical hepatectomy	23	(59)	183	(49)	0.257
Surgical duration, min^a^	368	(202–890)	334	(82–990)	0.109
Inflow occlusion time, min^a^	66	(15–150)	59	(0–247)	0.207
Blood loss, ml^a^	430	(25–2100)	278	(0–5500)	0.002
Intraoperative blood transfusion	10	(26)	51	(14)	0.065

Values in parentheses are percentages unless otherwise indicated.

Abbreviations: PHBL, post-hepatectomy bile leakage; HBsAg, hepatitis B surface antigen; HCVAb, hepatitis C virus antibody; Bil, bilirubin; AST, aspartate aminotransferase; ALT, alanine aminotransferase; PT, prothrombin time; AFP, alpha-fetoprotein; PIVKA-II, protein induced by vitamin K absence or antagonist II; ICGR15, indocyanine green retention rate at 15 min.

^a^Values are median (range).

### Preoperative muscle and fat areas

The differences in the muscle and fat areas between the two groups are shown in Table 2. At the L3 level, the psoas muscle area and VFA were significantly larger in the PHBL (+) group than in the PHBL (−) group (16.6 vs. 14.0 cm^2^; 135.3 vs. 96.8 cm^2^; *P* = 0.016 and 0.003, respectively). In contrast, the SMI values were comparable between the two groups. Although the ratios of patients with sarcopenia were comparable, the ratio of patients with sarcopenic obesity was significantly higher in the PHBL (+) group than in the PHBL (−) group (89.7% vs. 59.7%; *P* < 0.001).

**Table 2:** Histopathological findings.

Variables	PHBL (+) (*n* = 39)		PHBL (−) (*n* = 370)		*P* value
Liver cirrhosis^a^	13	(33)	115	(31)	0.774
Maximum tumor diameter, cm^b^	2.8	(0.9–15.0)	2.5	(0.1–17.0)	0.523
Number of tumors					0.097
Solitary	33	(85)	270	(73)	
Multiple	6	(15)	100	(27)	
Milan criteria					0.930
Within	30	(77)	280	(76)	
Beyond	9	(23)	87	(24)	
Histological grade^c^					0.542
GX	0	(0)	0	(0)	
G1	4	(10)	61	(17)	
G2	24	(62)	204	(55)	
G3	11	(28)	104	(28)	
G4	0	(0)	0	(0)	
Microvascular invasion^c^	7	(18)	101	(27)	0.191
Portal vein invasion	5	(13)	81	(22)	0.164
Hepatic vein invasion	3	(8)	36	(10)	0.672
Pathological AJCC staging^c^					0.071
Stage I	10	(26)	103	(28)	
Stage II	19	(48)	145	(39)	
Stage III	10	(26)	91	(25)	
Stage IV	0	(0)	31	(8)	
Residual tumor					0.293
R0	35	(90)	349	(94)	
R1	4	(10)	21	(6)	

Values in parentheses are percentages unless otherwise indicated.

Abbreviations: AJCC, American Joint Committee on Cancer.

^a^Fibrosis score of 4 using the new Inuyama classification.

^b^Values are presented as median (range).

^c^In accordance with the definition in the American Joint Committee on Cancer Staging Manual, 7th edition.

### Surgical outcomes

Among the total cohort, the median surgical duration time was 340 min (range: 82–990 min), the median inflow occlusion time was 60 min (range: 0–247 min), and the median intraoperative blood loss was 300 mL (range: 0–5500 mL). The surgical outcomes are shown in Table 1. No significant differences were observed between the two groups with regards to the type of hepatectomy, the ratio of primary hepatectomy, or the ratio of anatomical hepatectomy. There were also no significant differences in the ratios of medial sectionectomy and central bisectionectomy. Furthermore, the surgical duration and inflow occlusion times were comparable between the two groups. However, intraoperative blood loss was greater in the PHBL (+) group than in the PHBL (−) group (430 vs. 278 mL; *P* = 0.002). As a result, the percentage of intraoperative blood transfusions tended to be higher in the PHBL (+) group (25.6% vs. 13.8%; *P* = 0.065).

### Histopathological findings

The histopathological findings are summarized in Table 2. The prevalences of liver cirrhosis were comparable between the two groups (*P* = 0.074). In contrast, the ratio of multiple tumors tended to be lower in the PHBL (+) group than in the PHBL (−) group (15.4% vs. 27.0%; *P* = 0.097). Furthermore, the pathological AJCC staging tended to be lower in the PHBL (+) group (*P* = 0.071). There were no significant differences between the two groups in the maximum tumor diameter, the ratio of microvascular invasion, or the R0 resection rate.

### Short-term outcomes

Regarding the short-term outcomes (Table 3), 225 patients (55.0%) developed postoperative complications, including 70 (17.1%) with complications of grade III or higher [10]. One patient (0.2%) in the total cohort died due to grade C PHLF. In the PHBL (+) group, 12 patients (30.8%) developed grade B PHBL and 27 (69.2%) developed grade A PHBL, while no patients developed grade C PHBL. Regarding PHLF, all cases of PHLF in the PHBL group were grade A, and there was no significant difference in the occurrence of PHLF between the two groups. Moreover, the incidences of intra-abdominal infection and pleural effusion were comparable between the two groups. However, the postoperative hospital stay was longer in the PHBL (+) group (14 vs. 12 d; *P* = 0.036).

**Table 3:** Short-term outcomes.

Variables	PHBL (+) (*n* = 39)		PHBL (−) (*n* = 370)		*P* value
90-day Mortality	0	(0)	1	(0.2)	0.654
Morbidity	39	(100)	186	(50)	< 0.001
Clavien–Dindo classification ≥III	12	(31)	58	(16)	0.027
Post-hepatectomy bile leakage					< 0.001
Grade A	27	(69)	0	(0)	
Grade B	12	(31)	0	(0)	
Grade C	0	(0)	0	(0)	
Post-hepatectomy liver failure					0.321
Grade A	5	(13)	48	(13)	
Grade B	0	(0)	16	(4)	
Grade C	0	(0)	1	(1)	
Incisional surgical site infection	3	(8)	23	(6)	0.727
Intra–abdominal infection	4	(10)	15	(4)	0.122
Pleural effusion	7	(18)	37	(10)	0.156
Postoperative hospital stay, days ^a^	14	(7–130)	12	(4–117)	0.036

Values in parentheses are percentages unless otherwise indicated.

Abbreviations: PHBL, post-hepatectomy bile leakage.

^a^Values are median (range).

### Risk factors for PHBL

The cut-off value for intraoperative blood loss was determined using a ROC curve analysis. The independent risk factors for the occurrence of PHBL were intraoperative blood loss ≥370 mL (odds ratio [OR]: 2.25; 95% confidence interval [CI]: 1.10–4.59; *P* = 0.026) and sarcopenic obesity (OR: 4.08; 95% CI: 1.37–12.1; *P* = 0.011) (Table 4). Furthermore, when the analysis was restricted to patients who underwent primary hepatectomy, sarcopenic obesity (OR: 3.61; 95% CI: 1.21–10.8; *P* = 0.022) was the only independent risk factor for the occurrence of PHBL.

**Table 4:** Univariate and multivariate analyses of risk factors for the occurrence of post-hepatectomy bile leakage.

	Univariate analysis		Multivariate analysis	
Variable	Odds ratio (95% CI)	*P* value	Odds ratio (95% CI)	*P* value
Age, ≥ 70 years	1.02 (0.52–1.98)	0.956		
Sex, male	3.02 (1.05–8.73)	0.041	1.80 (0.18–1.68)	0.298
Body mass index, ≥ 25 kg/m^2^	1.14 (0.53–2.43)	0.737		
Hypertension, yes	2.57 (1.22–5.43)	0.013	1.80 (0.83–3.91)	0.138
Diabetes mellites, yes	1.15 (0.58–2.30)	0.687		
Surgical duration, ≥ 340 min	1.41 (0.73–2.74)	0.310		
Inflow occlusion time, ≥ 60 min	1.62 (0.82–3.18)	0.164		
Blood loss, ≥ 370 ml	3.00 (1.51–5.97)	0.002	2.25 (1.10–4.59)	0.026
Anatomical hepatectomy, yes	1.47 (0.75–2.87)	0.261		
Multiple tumors, yes	0.49 (0.20–1.21)	0.121		
Sarcopenia, yes	1.13 (0.58–2.21)	0.722		
Sarcopenic obesity, yes	5.90 (2.05–16.9)	0.001	4.08 (1.37–12.1)	0.011
Liver cirrhosis, yes	1.11 (0.55–2.24)	0.773		

Abbreviations: CI, confidence interval.

**Fig 2:**
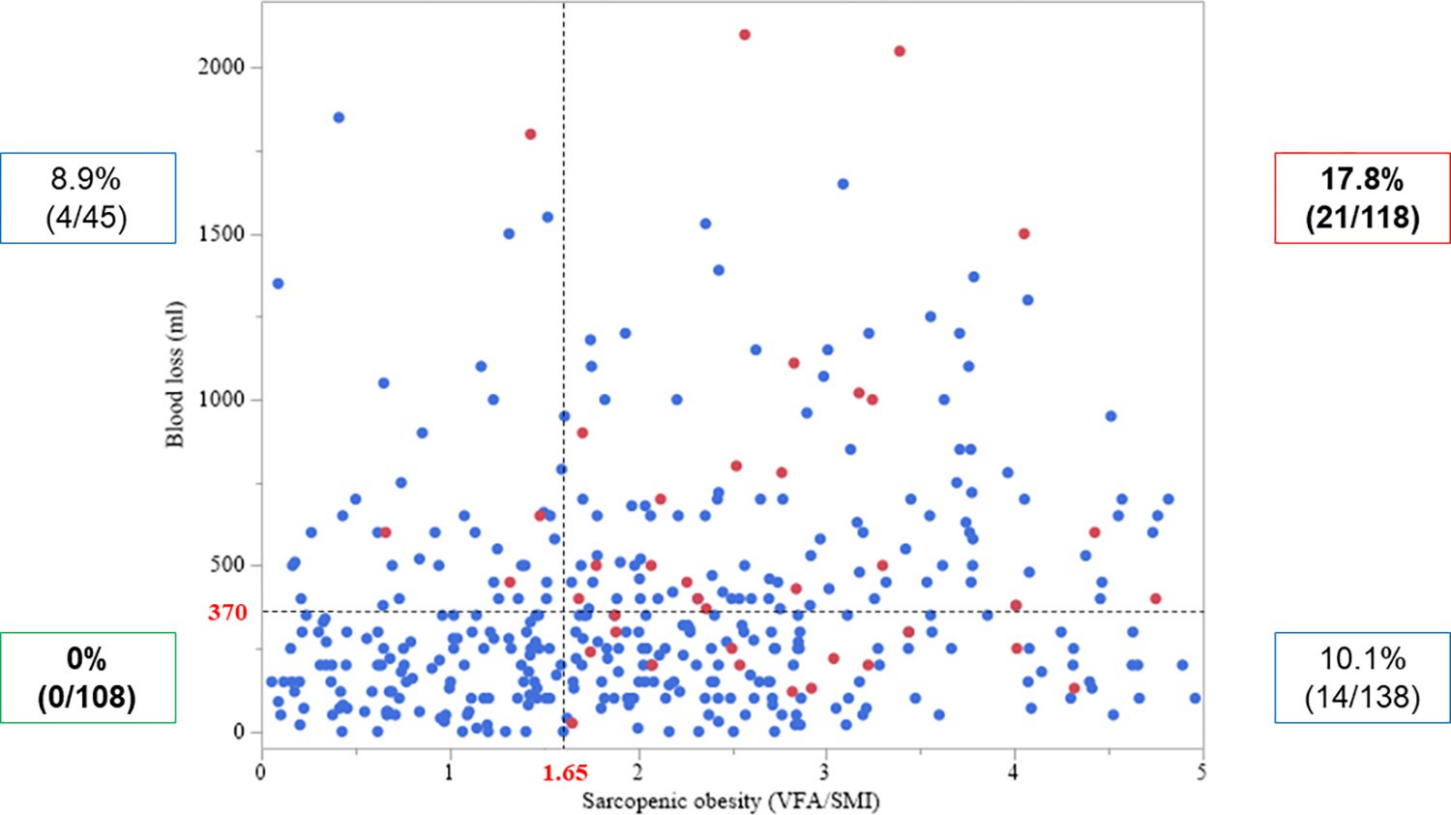
Scatter diagram of the correlation between visceral fat area (VFA)/skeletal muscle index (SMI) and intraoperative blood loss. Coefficients (ρ) and *P*-values were calculated using Spearman’s rank correlation analysis. Red circles, patients with post-hepatectomy bile leakage (PHBL); blue circles, patients without PHBL.

**Fig 3:**
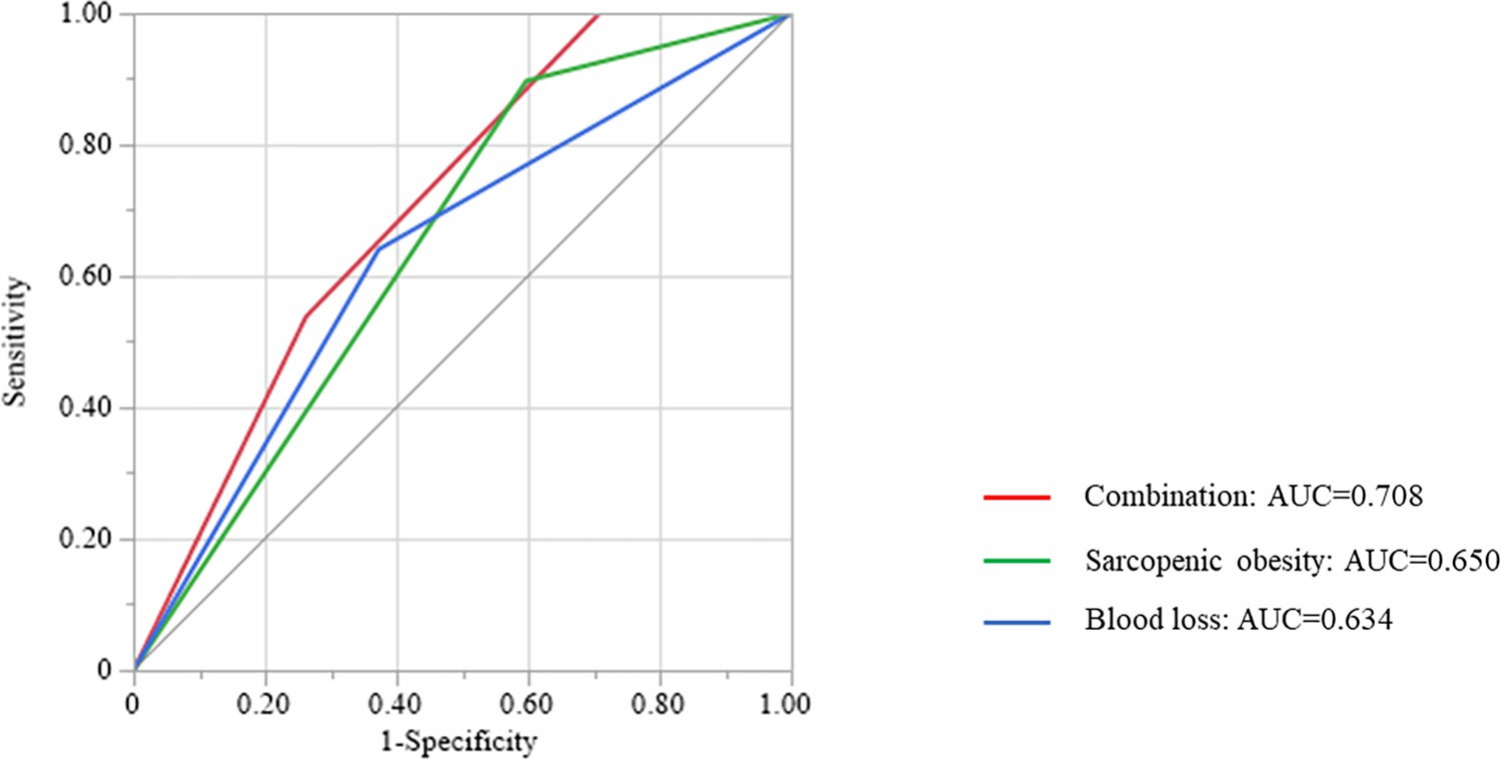
Receiver operating characteristic (ROC) curve analysis for the occurrence of post-hepatectomy bile leakage. Red, ROC curve of the combination of sarcopenic obesity and intraoperative blood loss; green, ROC curve of sarcopenic obesity; blue, ROC curve of intraoperative blood loss.

A scatter diagram of the correlation between intraoperative blood loss and sarcopenic obesity (VFA/SMI) was created (Fig 2). Spearman’s rank correlation analysis showed a low correlation between the amount of intraoperative blood loss and the VFA/SMI value (*ρ* = 0.201; *P* < 0.001). No patients with intraoperative blood loss <370 mL and without sarcopenic obesity developed PHBL (0 of 108 patients). In contrast, patients with intraoperative blood loss ≥370 mL and sarcopenic obesity had the highest ratio of the occurrence of PHBL among all combinations of these two parameters (17.8%; 21 of 118 patients). Moreover, a ROC curve analysis of combined intraoperative blood loss and sarcopenic obesity had a higher area under the curve value (0.708; *P* = 0.013) than either parameter alone (Fig 3).

## Discussion

Recently, the targets for hepatectomy have been expanding, to liver metastases of various carcinomas for example, and thus hepatectomy has been increasing in importance despite advances in drug therapy. PHBL is one of the most common postoperative complications after hepatectomy. Although most cases of PHBL can be resolved by conservative treatment, PHBL can lead to PHLF or sepsis from an infection. Shehta et al. [16] reported that PHBL occurred in 5.8% of hepatectomy patients, and their PHBL (+) group had higher grades of PHLF. Lo et al. [17] showed that biliary complications including PHBL developed in 8.1% of hepatectomy patients, and that these complications carried high risks for PHLF and mortality. In contrast, Okabayashi et al. [18] revealed that high body mass index (BMI), high intraoperative blood loss, presence of PHBL, and poor postoperative glucose control were risk factors for the occurrence of SSI in a multivariate analysis. In the present study, no significant differences were observed in the occurrences of PHLF and SSI between the PHBL (+) and PHBL (−) groups. These findings may be attributed to the close monitoring and early intervention and appropriate usage of prophylactic antibiotics at our institute. The results of many previous studies have indicated that PHBL is associated with other postoperative complications after hepatectomy; hence, it is desirable and reasonable to try and preoperatively identify the patients at a high risk for PHBL to improve their postoperative outcomes.

Obesity is one of the main health concerns that needs to be resolved, not only in Japan, but also in developed countries in particular. Obesity is clearly related to lifestyle diseases, such as hypertension, cardiovascular diseases, HCC from nonalcoholic steatohepatitis, and colorectal cancer [19]. A large database retrospective study [20] found that obesity was an independent risk factor for colorectal cancer across all age groups compared with the general population. BMI is the most common and simple indicator of obesity. Almost all previous reports that have shown a relationship between obesity and postoperative outcomes have been based on BMI. However, BMI can sometimes be inaccurate because it depends on muscle mass as well as fat mass. Therefore, we focused on sarcopenic obesity as a more objective and accurate indicator. Sarcopenic obesity was identified as an independent risk factor for the occurrence of PHBL in this study, whereas BMI was not.

Sarcopenic obesity, characterized by the depletion of lean body mass alongside the preservation or even augmentation of fat mass, serves as an indicator for assessment of patient nutrition [21]. Previous investigations suggested that sarcopenic obesity is a risk factor for both short- and long-term surgical outcomes. For example, Runkel et al. [22] reported that sarcopenic obesity was an independent risk factor for overall complications after hepatectomy for colorectal liver metastases. Regarding the association between fat and PHBL, our previous study [15] identified sarcopenic obesity as an independent risk factor for the occurrence of postoperative pancreatic fistula after pancreaticoduodenectomy. The rationale behind this observation was considered to arise from the potential complication posed by adipose tissue surrounding the pancreatic duct. The presence of this adipose tissue may complicate the anastomosis process, diminish the local blood flow essential for wound healing, and generate inflammatory cytokines that hinder the healing process. The surplus production of inflammatory cytokines by excessive adipose tissue has the potential to cause delayed healing of biliary wounds, consequently giving rise to the occurrence of PHBL. Another possible reason is that sarcopenic obesity may be associated with inadequate exposure to the incision of the liver. Underexposure of the operating field may affect the blood loss and bile leakage during the operation. Therefore, the results of the present study fit with existing data.

There are several reports on the relationship between intraoperative bleeding and PHBL. Wang et al. [23] found that tumor size, type of tumor, surgical duration time, blood loss, and blood transfusion were independent risk factors for PHBL. Intraoperative blood loss may cause liver damage and reduced blood flow around the bile duct, resulting in bile leakage. Another possibility is that surgery with a wide surface area for the incision exposes more bile ducts at the surface [24]. Hepatectomy with such a wide surface area of the incision may increase intraoperative blood loss, and the present findings may reflect this possibility. However, the present study was a retrospective study, and the area of the cut surface could not be measured accurately. Furthermore, as mentioned in the Results section, no significant difference was observed in the type of hepatectomy, such as medial sectionectomy or central bisectionectomy.

Occlusion of the hepatic inflow pedicle, also known as the PM, is a widely accepted method for reducing intraoperative blood loss. However, the PM results in ischemia-reperfusion changes [25]. According to several animal studies, ischemia-reperfusion injury caused by hilar vascular clamping can accelerate tumor growth, stimulate tumor cell adhesion, and promote metastasis [26]. Furthermore, studies have shown that PM duration for HCC was associated with postoperative long-term outcomes [27, 28]. However, because of recent advances in surgical techniques, the PM is not necessary for all operations. Maurer et al. [29] reported that major resection without the PM is feasible and safe and may reduce liver damage and failure. However, the decision to perform the PM during hepatectomy should be based on risk assessment and operative difficulties. Selection of the appropriate intraoperative supportive techniques required to complete the scheduled operation with minimal intraoperative blood loss is important.

Several limitations were present in this study. First, it was a single-center retrospective study, and thus the potential for selection bias exists. Second, the determined cut-off thresholds for sarcopenic obesity and intraoperative blood loss were established based on optimal sensitivities and specificities derived from ROC curve analyses for predicting PHBL. As a result, these cut-off values may not necessarily apply universally across different medical institutions. Notwithstanding these shortcomings, we maintain that our findings hold significance for surgeons because this study stands as a pioneering attempt to explore the correlation between sarcopenic obesity and PHBL in HCC patients.

## Conclusion

Sarcopenic obesity and intraoperative blood loss were identified as significant risk factors for the occurrence of PHBL. It is important to preoperatively understand whether a patient is at high or low risk for PHBL for early therapeutic intervention.

## Supporting information

S1 Checklist STROBE checklist. (DOCX)
