# Supplementary material for: Impact of sarcopenic obesity on post-hepatectomy bile leakage for hepatocellular carcinoma
Source: PLoS One. 2023 Oct 5;18(10):e0286353. doi: 10.1371/journal.pone.0286353 (PMC10553327; doi:10.1371/journal.pone.0286353)
Supplement: Attachment — Submitted filename: Response to Reviewers.docx [file pone.0286353.s002.docx]

Emily Chenette, PhD

Editor-in-Chief

*PLOS ONE*

17 August 2023

Dear Dr Chenette,

We are pleased to submit the first revised version of our manuscript “Impact of sarcopenic obesity on post-hepatectomy bile leakage for hepatocellular carcinoma” to *PLOS ONE*.

We have revised the original manuscript in accordance with the reviewers’ comments. Please find the following files attached:

• Response to Reviewers: Response letter including our point-by-point responses to the reviewers’ comments

• Revised Manuscript with Tracked Changes: Changes are highlighted in yellow

• Manuscript: An unmarked version of our revised paper

This manuscript has not been submitted for publication in any other journal. All coauthors have reviewed the revised manuscript and approved its resubmission. All coauthors declare no conflicts of interest.

Please address all correspondence to: Akira Shimizu, MD, PhD, Department of Surgery, Shinshu University School of Medicine, 3-1-1 Asahi, Matsumoto, Nagano 390-8621, Japan.

Phone: +81 263 37 2654; Fax: +81 263 35 1282

E-mail: ashimizu@shinshu-u.ac.jp

We hope that our paper will now be considered suitable for publication, and we look forward to hearing from you concerning your editorial decision.

Yours sincerely,

Akira Shimizu, MD, PhD

**Journal Requirements:**

**1. Please ensure that your manuscript meets PLOS ONE's style requirements, including those for file naming. The PLOS ONE style templates can be found at**

**https://journals.plos.org/plosone/s/file?id=wjVg/PLOSOne_formatting_sample_main_body.pdf and**

**https://journals.plos.org/plosone/s/file?id=ba62/PLOSOne_formatting_sample_title_authors_affiliations.pdf**

Response: We appreciate this comment. We have changed our manuscript to meet the style requirements of *PLOS ONE*.

**2. We noticed you have some minor occurrence of overlapping text with the following previous publication(s), which needs to be addressed:**

**- https://onlinelibrary.wiley.com/doi/10.1002/jhbp.1283**

**- https://www.sciencedirect.com/science/article/pii/S1015958422004651?via%3Dihub**

**In your revision ensure you cite all your sources (including your own works), and quote or rephrase any duplicated text outside the methods section. Further consideration is dependent on these concerns being addressed.**

Response: We appreciate this comment. Some text overlaps have been rephrased to the extent possible outside the Methods section.

**3. We note that you have indicated that data from this study are available upon request. PLOS only allows data to be available upon request if there are legal or ethical restrictions on sharing data publicly. For more information on unacceptable data access restrictions, please see http://journals.plos.org/plosone/s/data-availability#loc-unacceptable-data-access-restrictions.**

**In your revised cover letter, please address the following prompts:**

**a) If there are ethical or legal restrictions on sharing a de-identified data set, please explain them in detail (e.g., data contain potentially sensitive information, data are owned by a third-party organization, etc.) and who has imposed them (e.g., an ethics committee). Please also provide contact information for a data access committee, ethics committee, or other institutional body to which data requests may be sent.**

**b) If there are no restrictions, please upload the minimal anonymized data set necessary to replicate your study findings as either Supporting Information files or to a stable, public repository and provide us with the relevant URLs, DOIs, or accession numbers. For a list of acceptable repositories, please see http://journals.plos.org/plosone/s/data-availability#loc-recommended-repositories.**

**We will update your Data Availability statement on your behalf to reflect the information you provide.**

Response: We apologize for the description in our Data Availability statement. We have added the required information, and modified the statement in the revised manuscript as shown below.

**Page 22, lines 325–330 (revised manuscript):**

**Data Availability**

Data cannot be shared publicly from the point of view of personal information protection. Data are available from the Shinshu Institutional Data Access/Ethics Committee (contact via shinhp@shinshu-u.ac.jp) for researchers who meet the criteria for access to confidential data. The data underlying the results presented in the study are available from Shinshu University (shinhp@shinshu-u.ac.jp).

**4. We note that you have included the phrase “data not shown” in your manuscript. Unfortunately, this does not meet our data sharing requirements. PLOS does not permit references to inaccessible data. We require that authors provide all relevant data within the paper, Supporting Information files, or in an acceptable, public repository. Please add a citation to support this phrase or upload the data that corresponds with these findings to a stable repository (such as Figshare or Dryad) and provide and URLs, DOIs, or accession numbers that may be used to access these data. Or, if the data are not a core part of the research being presented in your study, we ask that you remove the phrase that refers to these data.**

Response: We apologize for using the phrase "data not shown". The data in question do not represent a core part of the research in the present study. Therefore, we have removed the phrase.

**5. Please include your tables as part of your main manuscript and remove the individual files. Please note that supplementary tables (should remain/ be uploaded) as separate “supporting information” files.**

Response: We apologize that our manuscript did not meet the style requirements of *PLOS ONE*. We have included our tables as part of the main manuscript and removed the individual files.

**6. Please review your reference list to ensure that it is complete and correct. If you have cited papers that have been retracted, please include the rationale for doing so in the manuscript text, or remove these references and replace them with relevant current references. Any changes to the reference list should be mentioned in the rebuttal letter that accompanies your revised manuscript. If you need to cite a retracted article, indicate the article’s retracted status in the References list and also include a citation and full reference for the retraction notice.**

Response: We appreciate this comment. We have checked references again, and have added one reference.

**Page 23, lines 347–349 (revised manuscript):**

5. Nakano R, Ohira M, Kobayashi T, Imaoka Y, Mashima H, Yamaguchi M, et al. Independent risk factors that predict bile leakage after hepatectomy for hepatocellular carcinoma: Cohort study. Int J Surg 2018;57:1-7.

**Reviewers' Comments to Author:**

**Reviewer #1: This retrospective study investigated the association between sarcopenic obesity and post-hepatectomy bile leakage. The authors found intraoperative blood loss ≥370 mL and sarcopenic obesity were independent risk factors for the occurrence of post-hepatectomy bile leakage.
The clinical significance of the present study is poor. Moreover, intraoperative blood loss ≥370 mL alwarys means major hepatectomy, which may cause post-hepatectomy bile leakage. Sarcopenic obesity is often indicative of inadequate exposure to the incision. Underexposure of the operating field often affects the examination of hemostasis and bile leakage during operation.**

Response: We greatly appreciate these insightful comments from Reviewer #1. As the reviewer commented, high intraoperative blood loss has the possibility to mean major hepatectomy, which may cause post-hepatectomy bile leakage. However, in the present study, the ratios of hemihepatectomy or more, major hepatectomy, and anatomical hepatectomy, and the surgical duration times were comparable between the two groups. This was a single-center retrospective study, and thus further studies are warranted to confirm the findings. Furthermore, as the reviewer mentioned, sarcopenic obesity is associated with the possibility of inadequate exposure to the incision of the liver. Underexposure of the operating field may affect the blood loss and bile leakage during the operation. We believed that the reviewer's comments are significant and should be addressed in the main text. Therefore, we have added some text to the revised manuscript as shown below.

**Page 19, lines 277–280 (revised manuscript):**

Another possible reason is that sarcopenic obesity may be associated with inadequate exposure to the incision of the liver. Underexposure of the operating field may affect the blood loss and bile leakage during the operation.

**Reviewer #2: This is a retrospective review looking at sarcopenic obesity as a risk factor for post-hepatectomy bile leak in patients undergoing resection for HCC. The overall manuscript is easy to read but will need minor grammatical/typographical editing. There are no tables shown in the submitted manuscript although the manuscript lists 4 tables in the text. The figures are appropriate and clear.**

Response: We thank the reviewer for these comments. We have submitted the revised manuscript to an English editing company to resolve any grammatical and typographical issues. We apologize for not including the tables in the main text, and have included the tables in the main text of the revised manuscript.

**Reviewer #3: This article studied Impact of sarcopenic obesity on post-hepatectomy bile leakage for hepatocellular carcinoma. The authors discovered Male gender, hypertension and cardiac disease, white blood cell counts, the psoas muscle area and visceral fat area, and intraoperative blood loss were significantly increased in the PHBL (+) group compared with the PHBL (−) group. Multivariate analysis showed that independent risk factors for the occurrence of PHBL were intraoperative blood loss ≥370 52 mL and sarcopenic obesity. The article had great clinical significance. But I didn't found the tables of this article.**

Response: We thank the reviewer for noting the clinical significance of our research. We apologize for not including the tables in the main text, and have included the tables in the main text of the revised manuscript.

**Reviewer #4: Bile leakage is an essential complication after hepatic surgery, however, it receives minimal attention by health care providers. The evaluation of the impact of sracopenia as an additive risk factor.
I enjoyed reviewing the manuscript and I think it worth publication in the repsected journal.**

Response: We thank the reviewer for these comments. We believe that the revisions made have helped to further improve our manuscript.
