# Supplementary material for: Impact of sarcopenic obesity on post-hepatectomy bile leakage for hepatocellular carcinoma
Source: PLoS One. 2023 Oct 5;18(10):e0286353. doi: 10.1371/journal.pone.0286353 (PMC10553327; doi:10.1371/journal.pone.0286353)
Supplement: Peer review history

# Peer review: Decision Letter 0

**DOI:** 10.1371/journal.pone.0286353.r001

**Authors:** Ezio Lanza

2 Aug 2023

PONE-D-23-14412Impact of sarcopenic obesity on post-hepatectomy bile leakage for hepatocellular carcinomaPLOS ONE

Dear Dr. Shimizu,

Thank you for submitting your manuscript to PLOS ONE. After careful consideration, we feel that it has merit but does not fully meet PLOS ONE’s publication criteria as it currently stands. Therefore, we invite you to submit a revised version of the manuscript that addresses the points raised during the review process.

==============================

Despite some mixed reviews, I feel that the manuscript has merit and should be considered for publication. Please amend the few remaining comments as below, mainly some grammatical errors. Consider using an online writing assistant to clean the whole manuscript if needed.  

For trasparency please provide with a response to Reviewer 1 comments. 

==============================

Please submit your revised manuscript by Sep 16 2023 11:59PM. If you will need more time than this to complete your revisions, please reply to this message or contact the journal office at plosone@plos.org. When you're ready to submit your revision, log on to https://www.editorialmanager.com/pone/ and select the 'Submissions Needing Revision' folder to locate your manuscript file.

Please include the following items when submitting your revised manuscript:

- A rebuttal letter that responds to each point raised by the academic editor and reviewer(s). You should upload this letter as a separate file labeled 'Response to Reviewers'.
- A marked-up copy of your manuscript that highlights changes made to the original version. You should upload this as a separate file labeled 'Revised Manuscript with Track Changes'.
- An unmarked version of your revised paper without tracked changes. You should upload this as a separate file labeled 'Manuscript'.

If you would like to make changes to your financial disclosure, please include your updated statement in your cover letter. Guidelines for resubmitting your figure files are available below the reviewer comments at the end of this letter.

If applicable, we recommend that you deposit your laboratory protocols in protocols.io to enhance the reproducibility of your results. Protocols.io assigns your protocol its own identifier (DOI) so that it can be cited independently in the future. For instructions see: https://journals.plos.org/plosone/s/submission-guidelines#loc-laboratory-protocols. Additionally, PLOS ONE offers an option for publishing peer-reviewed Lab Protocol articles, which describe protocols hosted on protocols.io. Read more information on sharing protocols at https://plos.org/protocols?utm_medium=editorial-email&utm_source=authorletters&utm_campaign=protocols.

We look forward to receiving your revised manuscript.

Kind regards,

Ezio Lanza, M.D.

Academic Editor

PLOS ONE

Journal Requirements:

When submitting your revision, we need you to address these additional requirements.

1. Please ensure that your manuscript meets PLOS ONE's style requirements, including those for file naming. The PLOS ONE style templates can be found at

https://journals.plos.org/plosone/s/file?id=wjVg/PLOSOne_formatting_sample_main_body.pdf and

https://journals.plos.org/plosone/s/file?id=ba62/PLOSOne_formatting_sample_title_authors_affiliations.pdf

2. We noticed you have some minor occurrence of overlapping text with the following previous publication(s), which needs to be addressed:

- https://onlinelibrary.wiley.com/doi/10.1002/jhbp.1283

- https://www.sciencedirect.com/science/article/pii/S1015958422004651?via%3Dihub

In your revision ensure you cite all your sources (including your own works), and quote or rephrase any duplicated text outside the methods section. Further consideration is dependent on these concerns being addressed.

3. We note that you have indicated that data from this study are available upon request. PLOS only allows data to be available upon request if there are legal or ethical restrictions on sharing data publicly. For more information on unacceptable data access restrictions, please see http://journals.plos.org/plosone/s/data-availability#loc-unacceptable-data-access-restrictions.

In your revised cover letter, please address the following prompts:

a) If there are ethical or legal restrictions on sharing a de-identified data set, please explain them in detail (e.g., data contain potentially sensitive information, data are owned by a third-party organization, etc.) and who has imposed them (e.g., an ethics committee). Please also provide contact information for a data access committee, ethics committee, or other institutional body to which data requests may be sent.

b) If there are no restrictions, please upload the minimal anonymized data set necessary to replicate your study findings as either Supporting Information files or to a stable, public repository and provide us with the relevant URLs, DOIs, or accession numbers. For a list of acceptable repositories, please see http://journals.plos.org/plosone/s/data-availability#loc-recommended-repositories.

We will update your Data Availability statement on your behalf to reflect the information you provide.4. We note that you have included the phrase “data not shown” in your manuscript. Unfortunately, this does not meet our data sharing requirements. PLOS does not permit references to inaccessible data. We require that authors provide all relevant data within the paper, Supporting Information files, or in an acceptable, public repository. Please add a citation to support this phrase or upload the data that corresponds with these findings to a stable repository (such as Figshare or Dryad) and provide and URLs, DOIs, or accession numbers that may be used to access these data. Or, if the data are not a core part of the research being presented in your study, we ask that you remove the phrase that refers to these data.

5. Please include your tables as part of your main manuscript and remove the individual files. Please note that supplementary tables (should remain/ be uploaded) as separate "supporting information" files.

6. Please review your reference list to ensure that it is complete and correct. If you have cited papers that have been retracted, please include the rationale for doing so in the manuscript text, or remove these references and replace them with relevant current references. Any changes to the reference list should be mentioned in the rebuttal letter that accompanies your revised manuscript. If you need to cite a retracted article, indicate the article’s retracted status in the References list and also include a citation and full reference for the retraction notice.

[Note: HTML markup is below. Please do not edit.]

Reviewers' comments:

Reviewer's Responses to Questions

**Comments to the Author**

1. Is the manuscript technically sound, and do the data support the conclusions?

The manuscript must describe a technically sound piece of scientific research with data that supports the conclusions. Experiments must have been conducted rigorously, with appropriate controls, replication, and sample sizes. The conclusions must be drawn appropriately based on the data presented. 

Reviewer #1: No

Reviewer #2: Yes

Reviewer #3: Yes

Reviewer #4: Yes

********** 

2. Has the statistical analysis been performed appropriately and rigorously? 

Reviewer #1: No

Reviewer #2: Yes

Reviewer #3: Yes

Reviewer #4: Yes

********** 

3. Have the authors made all data underlying the findings in their manuscript fully available?

The PLOS Data policy requires authors to make all data underlying the findings described in their manuscript fully available without restriction, with rare exception (please refer to the Data Availability Statement in the manuscript PDF file). The data should be provided as part of the manuscript or its supporting information, or deposited to a public repository. For example, in addition to summary statistics, the data points behind means, medians and variance measures should be available. If there are restrictions on publicly sharing data—e.g. participant privacy or use of data from a third party—those must be specified.

Reviewer #1: No

Reviewer #2: No

Reviewer #3: No

Reviewer #4: Yes

********** 

4. Is the manuscript presented in an intelligible fashion and written in standard English?

PLOS ONE does not copyedit accepted manuscripts, so the language in submitted articles must be clear, correct, and unambiguous. Any typographical or grammatical errors should be corrected at revision, so please note any specific errors here.

Reviewer #1: Yes

Reviewer #2: Yes

Reviewer #3: Yes

Reviewer #4: Yes

********** 

5. Review Comments to the Author

Please use the space provided to explain your answers to the questions above. You may also include additional comments for the author, including concerns about dual publication, research ethics, or publication ethics. (Please upload your review as an attachment if it exceeds 20,000 characters)

Reviewer #1: This retrospective study investigated the association between sarcopenic obesity and post-hepatectomy bile leakage. The authors found intraoperative blood loss ≥370 mL and sarcopenic obesity were independent risk factors for the occurrence of post-hepatectomy bile leakage.

The clinical significance of the present study is poor. Moreover, intraoperative blood loss ≥370 mL alwarys means major hepatectomy, which may cause post-hepatectomy bile leakage. Sarcopenic obesity is often indicative of inadequate exposure to the incision. Underexposure of the operating field often affects the examination of hemostasis and bile leakage during operation.

Reviewer #2: This is a retrospective review looking at sarcopenic obesity as a risk factor for post-hepatectomy bile leak in patients undergoing resection for HCC. The overall manuscript is easy to read but will need minor grammatical/typographical editing. There are no tables shown in the submitted manuscript although the manuscript lists 4 tables in the text. The figures are appropriate and clear.

Reviewer #3: This article studied Impact of sarcopenic obesity on post-hepatectomy bile leakage for hepatocellular carcinoma. The authors discovered Male gender, hypertension and cardiac disease, white blood cell counts, the psoas muscle area and visceral fat area, and intraoperative blood loss were significantly increased in the PHBL (+) group compared with the PHBL (−) group. Multivariate analysis showed that independent risk factors for the occurrence of PHBL were intraoperative blood loss ≥370 52 mL and sarcopenic obesity. The article had great clinical significance. But I didn't found the tables of this article.

Reviewer #4: Bile leakage is an essential complication after hepatic surgery, however, it receives minimal attention by health care providers. The evaluation of the impact of sracopenia as an additive risk factor.

I enjoyed reviewing the manuscript and I think it worth publication in the repsected journal. 

********** 

6. PLOS authors have the option to publish the peer review history of their article (what does this mean?). If published, this will include your full peer review and any attached files.

If you choose “no”, your identity will remain anonymous but your review may still be made public.

**Do you want your identity to be public for this peer review?** For information about this choice, including consent withdrawal, please see our Privacy Policy.

Reviewer #1: No

Reviewer #2: **Yes: **Prof London Lucien Ooi

Reviewer #3: No

Reviewer #4: No

**********

[NOTE: If reviewer comments were submitted as an attachment file, they will be attached to this email and accessible via the submission site. Please log into your account, locate the manuscript record, and check for the action link "View Attachments". If this link does not appear, there are no attachment files.]

While revising your submission, please upload your figure files to the Preflight Analysis and Conversion Engine (PACE) digital diagnostic tool, https://pacev2.apexcovantage.com/. PACE helps ensure that figures meet PLOS requirements. To use PACE, you must first register as a user. Registration is free. Then, login and navigate to the UPLOAD tab, where you will find detailed instructions on how to use the tool. If you encounter any issues or have any questions when using PACE, please email PLOS at figures@plos.org. Please note that Supporting Information files do not need this step.

# Author comment: Author response to Decision Letter 0

**DOI:** 10.1371/journal.pone.0286353.r002

18 Aug 2023

Journal Requirements:

1. Please ensure that your manuscript meets PLOS ONE's style requirements, including those for file naming. The PLOS ONE style templates can be found at

https://journals.plos.org/plosone/s/file?id=wjVg/PLOSOne_formatting_sample_main_body.pdf and

https://journals.plos.org/plosone/s/file?id=ba62/PLOSOne_formatting_sample_title_authors_affiliations.pdf

Response: We appreciate this comment. We have changed our manuscript to meet the style requirements of PLOS ONE.

2. We noticed you have some minor occurrence of overlapping text with the following previous publication(s), which needs to be addressed:

- https://onlinelibrary.wiley.com/doi/10.1002/jhbp.1283

- https://www.sciencedirect.com/science/article/pii/S1015958422004651?via%3Dihub

In your revision ensure you cite all your sources (including your own works), and quote or rephrase any duplicated text outside the methods section. Further consideration is dependent on these concerns being addressed.

Response: We appreciate this comment. Some text overlaps have been rephrased to the extent possible outside the Methods section.

3. We note that you have indicated that data from this study are available upon request. PLOS only allows data to be available upon request if there are legal or ethical restrictions on sharing data publicly. For more information on unacceptable data access restrictions, please see http://journals.plos.org/plosone/s/data-availability#loc-unacceptable-data-access-restrictions.

In your revised cover letter, please address the following prompts:

a) If there are ethical or legal restrictions on sharing a de-identified data set, please explain them in detail (e.g., data contain potentially sensitive information, data are owned by a third-party organization, etc.) and who has imposed them (e.g., an ethics committee). Please also provide contact information for a data access committee, ethics committee, or other institutional body to which data requests may be sent.

b) If there are no restrictions, please upload the minimal anonymized data set necessary to replicate your study findings as either Supporting Information files or to a stable, public repository and provide us with the relevant URLs, DOIs, or accession numbers. For a list of acceptable repositories, please see http://journals.plos.org/plosone/s/data-availability#loc-recommended-repositories.

We will update your Data Availability statement on your behalf to reflect the information you provide.

Response: We apologize for the description in our Data Availability statement. We have added the required information, and modified the statement in the revised manuscript as shown below.

Page 22, lines 325–330 (revised manuscript):

Data Availability

Data cannot be shared publicly from the point of view of personal information protection. Data are available from the Shinshu Institutional Data Access/Ethics Committee (contact via shinhp@shinshu-u.ac.jp) for researchers who meet the criteria for access to confidential data. The data underlying the results presented in the study are available from Shinshu University (shinhp@shinshu-u.ac.jp).

4. We note that you have included the phrase “data not shown” in your manuscript. Unfortunately, this does not meet our data sharing requirements. PLOS does not permit references to inaccessible data. We require that authors provide all relevant data within the paper, Supporting Information files, or in an acceptable, public repository. Please add a citation to support this phrase or upload the data that corresponds with these findings to a stable repository (such as Figshare or Dryad) and provide and URLs, DOIs, or accession numbers that may be used to access these data. Or, if the data are not a core part of the research being presented in your study, we ask that you remove the phrase that refers to these data.

Response: We apologize for using the phrase "data not shown". The data in question do not represent a core part of the research in the present study. Therefore, we have removed the phrase.

5. Please include your tables as part of your main manuscript and remove the individual files. Please note that supplementary tables (should remain/ be uploaded) as separate “supporting information” files.

Response: We apologize that our manuscript did not meet the style requirements of PLOS ONE. We have included our tables as part of the main manuscript and removed the individual files.

6. Please review your reference list to ensure that it is complete and correct. If you have cited papers that have been retracted, please include the rationale for doing so in the manuscript text, or remove these references and replace them with relevant current references. Any changes to the reference list should be mentioned in the rebuttal letter that accompanies your revised manuscript. If you need to cite a retracted article, indicate the article’s retracted status in the References list and also include a citation and full reference for the retraction notice.

Response: We appreciate this comment. We have checked references again, and have added one reference.

Page 23, lines 347–349 (revised manuscript):

5. Nakano R, Ohira M, Kobayashi T, Imaoka Y, Mashima H, Yamaguchi M, et al. Independent risk factors that predict bile leakage after hepatectomy for hepatocellular carcinoma: Cohort study. Int J Surg 2018;57:1-7.

Reviewers' Comments to Author:

Reviewer #1: This retrospective study investigated the association between sarcopenic obesity and post-hepatectomy bile leakage. The authors found intraoperative blood loss ≥370 mL and sarcopenic obesity were independent risk factors for the occurrence of post-hepatectomy bile leakage.

The clinical significance of the present study is poor. Moreover, intraoperative blood loss ≥370 mL alwarys means major hepatectomy, which may cause post-hepatectomy bile leakage. Sarcopenic obesity is often indicative of inadequate exposure to the incision. Underexposure of the operating field often affects the examination of hemostasis and bile leakage during operation.

Response: We greatly appreciate these insightful comments from Reviewer #1. As the reviewer commented, high intraoperative blood loss has the possibility to mean major hepatectomy, which may cause post-hepatectomy bile leakage. However, in the present study, the ratios of hemihepatectomy or more, major hepatectomy, and anatomical hepatectomy, and the surgical duration times were comparable between the two groups. This was a single-center retrospective study, and thus further studies are warranted to confirm the findings. Furthermore, as the reviewer mentioned, sarcopenic obesity is associated with the possibility of inadequate exposure to the incision of the liver. Underexposure of the operating field may affect the blood loss and bile leakage during the operation. We believed that the reviewer's comments are significant and should be addressed in the main text. Therefore, we have added some text to the revised manuscript as shown below.

Page 19, lines 277–280 (revised manuscript):

Another possible reason is that sarcopenic obesity may be associated with inadequate exposure to the incision of the liver. Underexposure of the operating field may affect the blood loss and bile leakage during the operation.

Reviewer #2: This is a retrospective review looking at sarcopenic obesity as a risk factor for post-hepatectomy bile leak in patients undergoing resection for HCC. The overall manuscript is easy to read but will need minor grammatical/typographical editing. There are no tables shown in the submitted manuscript although the manuscript lists 4 tables in the text. The figures are appropriate and clear.

Response: We thank the reviewer for these comments. We have submitted the revised manuscript to an English editing company to resolve any grammatical and typographical issues. We apologize for not including the tables in the main text, and have included the tables in the main text of the revised manuscript.

Reviewer #3: This article studied Impact of sarcopenic obesity on post-hepatectomy bile leakage for hepatocellular carcinoma. The authors discovered Male gender, hypertension and cardiac disease, white blood cell counts, the psoas muscle area and visceral fat area, and intraoperative blood loss were significantly increased in the PHBL (+) group compared with the PHBL (−) group. Multivariate analysis showed that independent risk factors for the occurrence of PHBL were intraoperative blood loss ≥370 52 mL and sarcopenic obesity. The article had great clinical significance. But I didn't found the tables of this article.

Response: We thank the reviewer for noting the clinical significance of our research. We apologize for not including the tables in the main text, and have included the tables in the main text of the revised manuscript.

Reviewer #4: Bile leakage is an essential complication after hepatic surgery, however, it receives minimal attention by health care providers. The evaluation of the impact of sracopenia as an additive risk factor.

I enjoyed reviewing the manuscript and I think it worth publication in the repsected journal. 

Response: We thank the reviewer for these comments. We believe that the revisions made have helped to further improve our manuscript.

Attachment Submitted filename: Response to Reviewers.docx Click here for additional data file.

# Editor report: Decision Letter 1

**DOI:** 10.1371/journal.pone.0286353.r003

**Authors:** Ezio Lanza

18 Sep 2023

Impact of sarcopenic obesity on post-hepatectomy bile leakage for hepatocellular carcinoma

PONE-D-23-14412R1

Dear Dr. Shimizu,

We’re pleased to inform you that your manuscript has been judged scientifically suitable for publication and will be formally accepted for publication once it meets all outstanding technical requirements.

Within one week, you’ll receive an e-mail detailing the required amendments. When these have been addressed, you’ll receive a formal acceptance letter and your manuscript will be scheduled for publication.

An invoice for payment will follow shortly after the formal acceptance. To ensure an efficient process, please log into Editorial Manager at http://www.editorialmanager.com/pone/, click the 'Update My Information' link at the top of the page, and double check that your user information is up-to-date. If you have any billing related questions, please contact our Author Billing department directly at authorbilling@plos.org.

If your institution or institutions have a press office, please notify them about your upcoming paper to help maximize its impact. If they’ll be preparing press materials, please inform our press team as soon as possible -- no later than 48 hours after receiving the formal acceptance. Your manuscript will remain under strict press embargo until 2 pm Eastern Time on the date of publication. For more information, please contact onepress@plos.org.

Kind regards,

Ezio Lanza, M.D.

Academic Editor

PLOS ONE

# Editor report: Acceptance letter

**DOI:** 10.1371/journal.pone.0286353.r004

**Authors:** Ezio Lanza

26 Sep 2023

PONE-D-23-14412R1 

Impact of sarcopenic obesity on post-hepatectomy bile leakage for hepatocellular carcinoma 

Dear Dr. Shimizu:

I'm pleased to inform you that your manuscript has been deemed suitable for publication in PLOS ONE. Congratulations! Your manuscript is now with our production department. 

If your institution or institutions have a press office, please let them know about your upcoming paper now to help maximize its impact. If they'll be preparing press materials, please inform our press team within the next 48 hours. Your manuscript will remain under strict press embargo until 2 pm Eastern Time on the date of publication. For more information please contact onepress@plos.org.

If we can help with anything else, please email us at plosone@plos.org. 

Thank you for submitting your work to PLOS ONE and supporting open access. 

Kind regards, 

PLOS ONE Editorial Office Staff

on behalf of

Dr. Ezio Lanza 

Academic Editor

PLOS ONE
